# The Late Sir William Gowers, M.D., F.R.S.

**Published:** 1915-05-15

**Authors:** B. Burford Rawlings


### THE LATE SIR WILLIAM GOWERS, M.D., F.R.S.

By B. BURFORD RAWLINGS.

By the death of Sir William Gowers the hospital world loses not only a great physician and a great medical teacher, but a man whose views respecting hospital administration were marked by an almost unique freedom from professional prejudice.

The hospital physician lives hard by the fount of things, and is expected to be equipped not only with advanced knowledge, but with the original and creative powers which justify his claim to rank as a consultant. Hence the dominant position he customarily occupies is rarely in question: his word is law.

No one could be more insistent when needful upon his professional prerogatives than Sir William Gowers; but, while absolutely loyal to the rightful claims of his profession, he was quick to recognise all that was due to lay authority in that complex scheme of medical relief for the poor which makes our hospitals to depend for their maintenance upon voluntary offerings, and to perform with almost equal assiduity the part of the philanthropist and the teacher. Unlike many of his fellows, Sir William recognised two outstanding truths. First, that to ensure an adequate flow of subscriptions the inducement of reality in the lay government is indispensable; hence, he always advocated a strong lay board; and, secondly, that the manifest and avowed primary object of the hospital must be to benefit the patients in its care.

From his earliest professional years he was attached to the Queen Square Hospital, and had a deep and enduring affection for the institution whose child he claimed to be. Perhaps nobody was more painfully concerned about the dispute which in the early days of this century brought such irremediable injury to the hospital; and it is beyond question that had Sir William's views prevailed with his colleagues, there would have been no conflict between the medical and lay authorities.

If the birth of Dr. Gowers' reputation and the success of his career were due largely to his association with the National Hospital and its unparalleled staff, of which he became one of the most brilliant members, not less certainly was the hospital's eminence as a teacher the outcome in great measure of his own valuable work. Whether as writer or speaker, Sir William was gifted with a power and precision of language and fullness of perception which made his lectures and even his casual conversations attractive and illuminating. To press or elucidate a point he would address himself to a single listener as to a critical assembly, with an emphasis almost disconcerting, and nobody could exchange views with him upon a subject where he felt seriously without conviction of his sincerity. To secure the well-being and permanence of the voluntary system few things could be more effective than the presence among the medical workers of men like Sir William Gowers.

